# A Bioinformatics Analysis Reveals a Group of MocR Bacterial Transcriptional Regulators Linked to a Family of Genes Coding for Membrane Proteins

**DOI:** 10.1155/2016/4360285

**Published:** 2016-06-30

**Authors:** Teresa Milano, Sebastiana Angelaccio, Angela Tramonti, Martino Luigi Di Salvo, Roberto Contestabile, Stefano Pascarella

**Affiliations:** ^1^Dipartimento di Scienze Biochimiche, Sapienza Università di Roma, 00185 Roma, Italy; ^2^Istituto di Biologia e Patologia Molecolari, Consiglio Nazionale delle Ricerche, 00185 Roma, Italy

## Abstract

The MocR bacterial transcriptional regulators are characterized by an N-terminal domain, 60 residues long on average, possessing the winged-helix-turn-helix (wHTH) architecture responsible for DNA recognition and binding, linked to a large C-terminal domain (350 residues on average) that is homologous to fold type-I pyridoxal 5′-phosphate (PLP) dependent enzymes like aspartate aminotransferase (AAT). These regulators are involved in the expression of genes taking part in several metabolic pathways directly or indirectly connected to PLP chemistry, many of which are still uncharacterized. A bioinformatics analysis is here reported that studied the features of a distinct group of MocR regulators predicted to be functionally linked to a family of homologous genes coding for integral membrane proteins of unknown function. This group occurs mainly in the Actinobacteria and Gammaproteobacteria phyla. An analysis of the multiple sequence alignments of their wHTH and AAT domains suggested the presence of specificity-determining positions (SDPs). Mapping of SDPs onto a homology model of the AAT domain hinted at possible structural/functional roles in effector recognition. Likewise, SDPs in wHTH domain suggested the basis of specificity of Transcription Factor Binding Site recognition. The results reported represent a framework for rational design of experiments and for bioinformatics analysis of other MocR subgroups.

## 1. Introduction

The members of the GntR family of bacterial transcriptional regulators are characterized by the presence of two domains [1]. The N-terminal domain, 60 residues long on average, displays the winged-helix-turn-helix (wHTH) architecture and is responsible for DNA recognition and binding [2]. The C-terminal domain belongs to at least four different structural families and is essential for oligomerization and effector binding. The MocR [3, 4] subfamily of the GntR regulators (often denoted in the literature as GabR/MocR) is characterized by a large C-terminal domain (350 residues on average), whose structure is similar to fold type-I pyridoxal 5′-phosphate (PLP) dependent enzymes [5]. Aspartate aminotransferase (AAT) [6] is the archetypal enzyme representing this fold. The wHTH and AAT domains are connected to each other by a peptide linker of different lengths in different MocRs. The solution of the first three-dimensional structure of a MocR, named GabR, from* Bacillus subtilis *[7, 8] confirmed the presence of a C-terminal fold type-I domain and provided fundamental insights for further investigations aimed at deciphering the mechanism of action of these regulators.

The MocR regulators are widespread among eubacteria [2, 4] and can occur within different species as single orthologous or multiple paralogous genes. The members of this complex subfamily are possibly involved in the regulation of the expression of proteins taking part in several metabolic pathways, directly or indirectly connected to PLP chemistry, many of which are still uncharacterized. Since the MocR discovery, studies have been intensified and in several cases the regulons under the control of MocR regulators have been experimentally determined and characterized, although many details of their action mechanism remain obscure. For example, a member of MocR subfamily was named TauR since it activates the expression of taurine utilization genes in* Rhodobacter capsulatus *[9];* Bacillus subtilis* GabR [10] in the presence of PLP and *γ*-amino butyric acid (GABA) activates transcription of* gabT* and* gabD* encoding GABA aminotransferase and succinic semialdehyde dehydrogenase, respectively; PdxR is involved in the regulation of the PLP synthesis in several bacteria such as* Corynebacterium glutamicum *[11],* Streptococcus pneumoniae *[12],* Listeria monocytogenes *[13],* Streptococcus mutans *[14], and* Bacillus clausii *[15]. Recently, a new* Brevibacillus brevis* MocR member has been demonstrated to activate the expression of the gene coding for the enzyme d-alanyl-d-alanine ligase [16].

The C-terminal domain displays a highly variable sequence within the MocR subfamily [17] possibly reflecting variation of specificity for different effector molecules. For example, PdxR regulators respond to PLP binding [13, 15], while GabR regulators respond to PLP and GABA [18]. The structural and functional heterogeneity of the MocR subfamily suggests that different structural subgroups of the C-terminal domain may exist that specifically recognize different effectors. Within Firmicutes, it has been shown that the C-terminal domain of MocRs can be classified into at least three subgroups, each characterized by residue conservation at specific sequence positions, although no functional association with genes or metabolic pathways could be convincingly anticipated [17].

We provide here a bioinformatics analysis of the features distinguishing a group of MocR regulators predicted to be functionally linked to a family of homologous genes coding for integral membrane proteins of unknown function. An attempt to relate specifically conserved residues in the wHTH and AAT domains to function is also reported.

## 2. Materials and Methods

The reference RegPrecise database version 3.3 [19] was utilized to retrieve MocR sequences predicted to regulate expression of specific regulons. Amino acid sequences were collected from Uniprot [20] and RefSeq [21] databanks. RefSeq codes were utilized throughout our work. Syntenic genes were found in the SynTax databank [22]. Databank sequence searches utilized the BLAST suite [23]. Amino acid sequence alignments were carried out using MUSCLE [24] or ClustalO [25] programs, while they were displayed and edited using the Jalview software [26]. Sequence redundancy was eliminated with the program Cd-Hit [27]; tree calculation was performed using MEGA5.2 [28]. Prediction of specificity-determining positions (SDPs) [29], namely, the positions within a multiple sequence alignment specific of a protein subgroup, and sequence clustering have exploited the software JDet [30] along with the bundled programs Xdet [31] and S3det [32]. Homology modelling relied on Modeller [33] and PyMod [34] while molecular graphics on PyMOL (The PyMOL Molecular Graphics System, version 1.7.4, Schrödinger, LLC).

For comparative analysis of genomic regions, we excluded closely related strains; that is, if more than one regulon from different strains of the same bacterium was present in regulon dataset, only one of them was selected for further analysis (if available, the relative RefSeq genome was chosen). Genes encoding MocR proteins were localized in their respective genomes, and the intergenic regions putatively involved in the control of selected regulons were retrieved from NCBI GenBank [35]. For* de novo *identification of candidate Transcription Factor Binding Sites (TFBSs) in the training set of potential upstream region, the MEME web server [36] was used. A search for a DNA pattern of length from 5 to 8 bp (on the basis of the experimentally characterized MocR TFBSs), directly or inversely repeated more than once for each sequence, was carried out within putative promoter regions. Motifs were further validated by the construction of multiple alignments of orthologous DNA fragments, using the PRO-COFFEE method [37], specifically designed for promoter regions. Sequence logos for the derived DNA motifs were drawn using WebLogo [38] and compared to each other. The promoter regions were analyzed using the BProm tool [39] to detect the canonical -35 and canonical -10 motifs, in order to classify them as* bona fide* promoter elements. Candidate DNA-binding sites were submitted to the TOMTOM and FIMO tools of the MEME suite [36]. TOMTOM compares TFBSs with the known motifs contained in the databases Prodoric release 8.9 [40] and RegTransBase release version 7 [41] and displays the alignments to significant matches (*E*-value threshold < 0.01). With the aim of checking whether the TFBSs are present in single copy through the genomes, three reference genomes (one genome for each taxonomical division) were selected and FIMO was used to scan their upstream sequence elements for individual matches for each motif.

## 3. Results 

### 3.1. Sequence Alignment and Clustering

The databank RegPrecise version 3.3 (http://regprecise.lbl.gov/RegPrecise/index.jsp) was queried to find all the regulons predicted to be under the control of a regulator of the MocR family. The regulons are organized in the databank according to taxonomy, orthology, and similarity of the TFBSs. A subset of MocR regulons includes genes coding for hypothetical membrane proteins of unknown function denoted as YczE (Conserved Domain Database code COG2364). These membrane proteins share with the family YitT (InterPro family IPR003740, Pfam PF02588, and Conserved Domain COG1284) the presence of one or two DUF161 domains. Indeed, YczE and YitT proteins are generically annotated as “membrane proteins containing the DUF161 domain.” The YczE proteins are characterized by the absence of the C-terminal domain DUF2179 (corresponding to the PDB structure 3HLU) present in the members of the YitT family. The sequences of all the MocRs predicted to regulate YczE genes, to which we will refer as YczR, were collected (Table 1). Most regulons were from Actinobacteria; a few were found in Alpha- and Gammaproteobacteria (Table 1). The MocR and YczE-coding genes predicted by RegPrecise to be in the same regulon are divergently transcribed. The MocRs of this set of regulons will be referred to as “YczR RegPrecise” set throughout this work.

**Figure 1:**
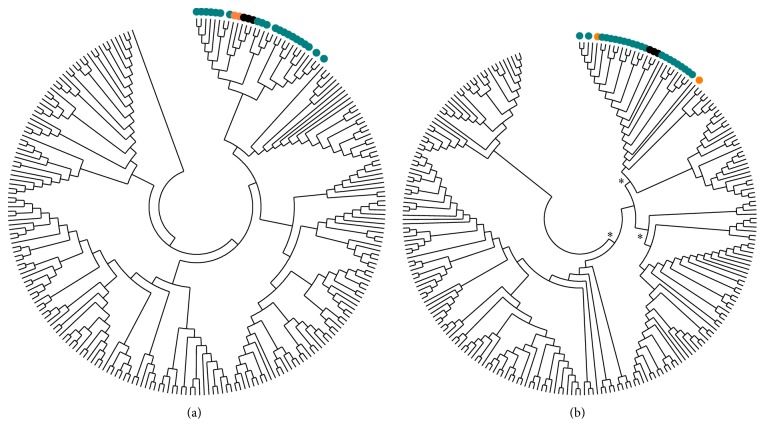
Cladogram of (a) the wHTH and (b) AAT domains of the MocR regulators from the RegPrecise databank. The cladograms have been calculated with the UPGMA method. Pairwise distances between sequences were calculated in units of number of amino acid differences. The bootstrap tree was inferred from 1000 replicates. Domains from the YczR predicted to be linked to the YczE genes are denoted by green (Actinobacteria), orange (Gammaproteobacteria), and black (Alphaproteobacteria) dots. Other taxa are from non-YczE MocRs. Asterisks denote nodes supported by at least 90% bootstrap frequency.

To verify whether the YczR RegPrecise set represented a real distinct group within the MocR family, all the MocR regulators not involved in YczE gene-containing regulons (MocR/other) were collected from the Actinobacteria and Gammaproteobacteria sets contained in the RegPrecise databank (Supplementary Information Table S1 in Supplementary Material available online at http://dx.doi.org/10.1155/2016/4360285). All the sequences (YczR RegPrecise and MocR/other) were retrieved from the RefSeq databank and multiply aligned with Muscle or ClustalO programs. The wHTH and AAT domains were subsequently analyzed separately. The UPGMA method in MEGA5.2 was applied to calculate a cladogram of the aligned sequences of the MocR domains (Figure 1). The cladograms clearly suggest that both domains of the YczR RegPrecise set cluster in the same subtree (Figure 1), irrespectively of their taxonomic origin. However, the bootstrap test does not support the inner nodes of the cladogram built for the wHTH domain (Figure 1(a)); the tree is nonetheless suggestive of plausible clustering. Moreover, this clustering is corroborated by the fact that it was independently reproduced by JDet analysis* (vide infra)*.

**Figure 2:**
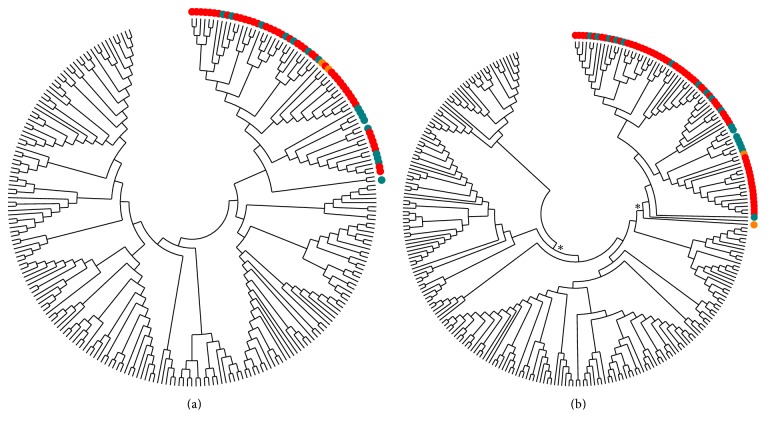
Cladogram of (a) the wHTH and (b) AAT domains of the MocR from RegPrecise and SynTax databanks. The cladograms have been calculated with the UPGMA method applied to multiple alignments of domain sequences filtered at 90% sequence identity. Pairwise distances between sequences were calculated in units of number of amino acid differences. The bootstrap tree was inferred from 1000 replicates. Domains from the YczR predicted to be linked to the YczE genes are denoted by green (Actinobacteria), orange (Gammaproteobacteria), and black (Alphaproteobacteria) dots. Red dots denote the YczR SynTax set. Other taxa are from non-YczE MocRs. Asterisks denote nodes supported by at least 90% bootstrap frequency.

The MocRs of other species sharing YczE synteny with those retrieved from RegPrecise databank were collected from the SynTax database (http://archaea.u-psud.fr/SyntTax/). Indeed, presence of synteny is another strong hint of functional association between the regulator and the adjacent divergently transcribed gene [42]. The list of the YczR genes from SynTax is reported in Supplementary Information Table S2. We will refer to this group of genes as “YczR SynTax” set. In the set, 55 regulators are from Gammaproteobacteria, 86 from Actinobacteria, and only 3 from Alphaproteobacteria. Multiple sequence alignments of wHTH and AAT domains from the YczR RegPrecise, MocR/other, and YczR SynTax were calculated. We will refer to the set of combined sequences YczR RegPrecise and YczR SynTax as “YczR” while to the set YczR plus MocR/other as “MocR_ALL”. To eliminate possibly confounding redundancy, the sequences of the MocR_ALL alignment were filtered at 90% sequence identity with the software Cd-Hit. Finally, a cladogram was built with MEGA5.2 using UPGMA clustering. The populations of YczR wHTH and AAT domains from RegPrecise and SynTax databanks cluster in the same subtree (Figure 2) although similar considerations about node statistical significance reported above for Figure 1(a) apply to Figure 2(a) as well. Nonetheless, the UPGMA trees strongly support the notion that both domains are conjointly subject to structural constraints during evolution.

### 3.2. Identification of SDPs and Mapping onto Homology Models

Potential SDPs of the YczR group were identified by application of the software JDet and the bundled programs Xdet and S3det to the multiple sequence alignments of the MocR_ALL wHTH and AAT domains filtered at 90% sequence identity. Average percentage of identity in the final sequence set was 30%.

S3det utilizes a vectorial representation of the sequences as a mean to cluster sequences on the base of their sequence similarities. In the unsupervised mode, the S3det algorithm is able to autonomously define subfamilies in a set of sequences and locate the residues that uniquely characterize each group, namely, the Specificity-Determining Positions (SDPs). Xdet program spots the SDPs by examining the correlated mutational behavior of the positions characteristic of a subfamily. Identification of SDPs however is not a trivial task and the methods available are still poorly performing [43]. Quality of the multiple sequence alignment is a factor strongly influencing the identification of the significant positions.

**Figure 3:**
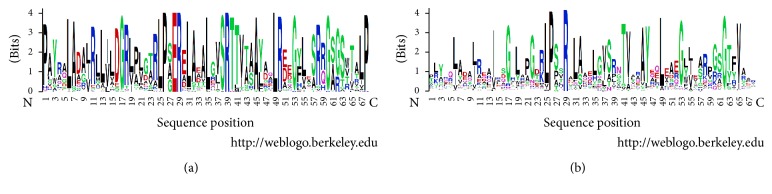
Logos of the alignments of the wHTH domain sequences from (a) YczR and (b) MocR/other. Logos were calculated by the site WebLogo. Residues are represented with the one-letter code. *x*-axis indicates sequence position. The overall height of each letter stack indicates the sequence conservation at that position, while the height of symbols within the stack indicates the relative frequency of each amino or nucleic acid at that position. Colors reflect chemical-physical residue properties.

**Figure 4:**
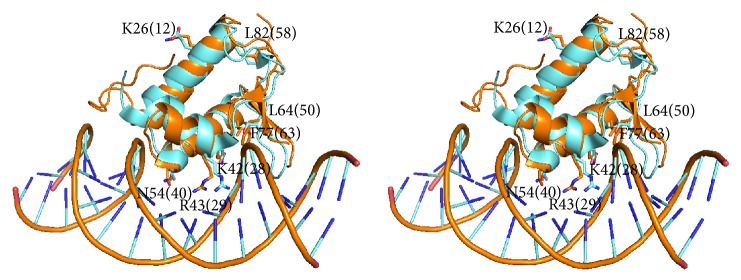
Stereo superposition between the wHTH domains of GabR from* Bacillus subtilis* and FadR from* E. coli*. Stereo superposition between the wHTH domains of GabR from* Bacillus subtilis* (orange cartoon, PDB code 4TV7) and of FadR from* E. coli* (cyan, PDB code 1H9T). GabR and FadR residues discussed in the text are displayed as sticks. Labels denote GabR residues with amino acid single letter code. Numbering refers to GabR structure and to Figure 3 (in parentheses). Table 2 reports the correspondence between the residues and their numbering. In particular, Arg29 corresponds to GabR Arg43 and FadR Arg35. DNA phosphate backbone is displayed as orange wire while bases are depicted with cyan and blue sticks.

Application of S3det analysis to the wHTH domains in the alignment of the sequence set MocR_ALL filtered at 90% sequence identity was able to independently reproduce the clustering obtained by UPGMA and reported in Figure 2(a). Alignment of the wHTH domains was unambiguous because of the presence of only very few and short indels (Supplementary Information Figure S1). The JDet analysis indicated as SDP characteristic of the wHTH domains of the YczR group the residue Glu28 (Figure 3). The same position is occupied in the other MocRs by a variety of polar and apolar residues (Figure 3). Comparison of the logos calculated for the wHTH domains of the YczR set and for its complement MocR/other set suggests that other residues may be characteristic of the former group, namely, Leu12, Thr40, Arg50, Ser63, and Pro68 (Figures 3 and 4). These residues were mapped onto the corresponding positions of the wHTH domain of the GabR regulator whose three-dimensional structure has been solved. Glu28 and Arg50 are equivalent to GabR Lys42 and Leu64, respectively (Table 2). Unfortunately, no three-dimensional structure of a GabR-DNA complex has been solved yet. However, the structure of a GntR regulator of the FadR family in complex with DNA has been deposited in the PDB with code 1H9T [44]. This regulator, involved in the regulation of fatty acid metabolism, possesses a wHTH domain homologous to the corresponding MocR domain. Using the wHTH domain of 1H9T as a template for modelling DNA interaction and recognition, one can infer that GabR Lys42 (and, by transitive property, YczR Glu28) is involved in the interaction with the phosphate backbone (Figure 4). Likewise, YczR Arg50 replacing GabR Leu64 may also interact with the phosphate scaffold (Table 2). All these inferences, however, should be considered with great caution due to the variability of the wHTH domain sequences from different species.

**Figure 5:**
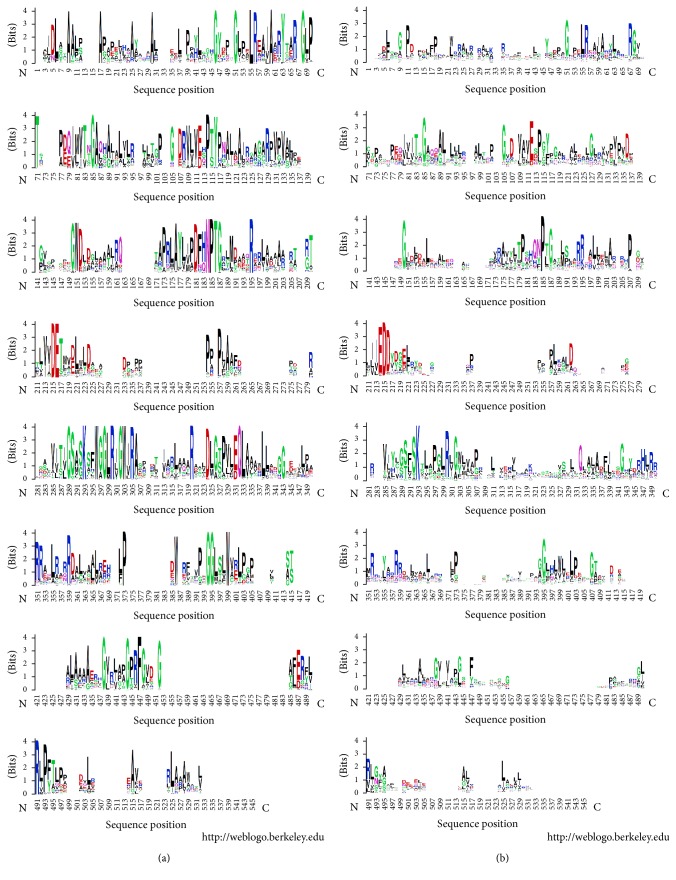
Logos of the alignments of the AAT domain sequences. Logos of the alignments of the AAT domain sequences from (a) YczR and (b) MocR/other. Logos were calculated by the site WebLogo. Letters and colors have the meaning described in the caption of Figure 3.

**Figure 6:**
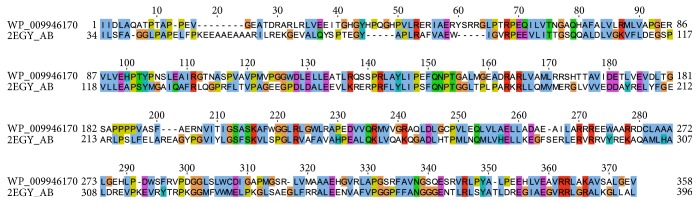
Sequence alignment between AAT domains for homology modelling. Sequence alignment between the AAT domains of the MocR from* Saccharopolyspora erythraea* (RefSeq code WP_009946170) and the template *α*-aminoadipate aminotransferase from* Thermus thermophilus* (PDB code 2EGY).

**Figure 7:**
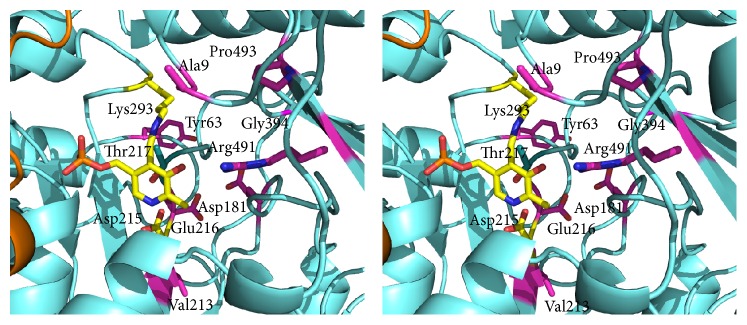
Stereo picture of the active site of the model of the AAT domain. Stereo picture of the active site of the model of the AAT domain of the MocR from* Saccharopolyspora erythraea* (RefSeq code WP_009946170). Pyridoxal phosphate, Lys forming the internal aldimine, and the Asp interacting with the pyridine nitrogen atom are represented as yellow stick models. Residues indicated as SDPs are colored in magenta. Numbering system refers to Figure 5.

Identification of SDPs specific of the AAT domain of YczR group is dependent on the alignment and particularly on the positioning of indels. AAT domains contain several indels that compound the calculation of the multiple alignments (Supplementary Information Figure S2). For that reason, we calculated two multiple alignments of the same sequence set with the programs ClustalO and Muscle and applied a consensus criterion: we considered* bona fide* SDPs those positions that were predicted by Xdet and S3det in one alignment and by one of the two methods in the other. For example, if position A was predicted as SDP by Xdet and S3det in the alignment calculated by ClustalO and by S3det in the Muscle alignment, then A would be considered an authentic SDP. If position B was predicted as SDP only by S3det in the alignment calculated by ClustalO and S3det in the Muscle alignment, then B would not be considered an SDP. S3det was able to reproduce the clustering reported in Figure 2(b). However, S3det identified two clusters in the alignment obtained with ClustalO and three (one of which coinciding with the YczR group) in the other calculated with Muscle. The residues responding to these criteria are (according to the numbering system of Figure 5 reporting the logos calculated separately for the AAT domains of YczR and MocR/other) Ala9, Tyr63, Asp181, Val 213, Glu216, Arg341, Gly394, and Pro493. In order to hypothesize a possible functional role for these residues, they were mapped onto the homology model built for a representative AAT domain of the YczR set. The model of the AAT domain of the MocR from* Saccharopolyspora erythraea* (RefSeq code WP_009946170) was built on the template structure *α*-aminoadipate aminotransferase from* Thermus thermophilus* (PDB code 2EGY) with which it shares 33% sequence identity (alignment displayed in Figure 6). This target-template pair was chosen after a BLAST search of the AAT domains of the YczR sequences over the PDB databank because it displayed one of the highest sequence similarities. Indeed, the target shared only about 20% sequence identity with the AAT domain of GabR regulator.  Table 3 reports possible structural roles for these residues gathered by the inspection of the homology model while Figure 7 displays the three-dimensional structure of the model and the relevant residues. It should be noted that the multiple sequence alignment (Supplementary Information Figure S2) and the structural model display the presence of the conserved residues Asp215 and Lys293 (numbering refers to Figure 5) corresponding to those that, in AAT and in the structural template 2EGY, interact with the pyridine nitrogen of the PLP and form the internal aldimine, respectively. For that reason, we assumed that YczR regulators have retained the potentiality to bind the PLP molecule. In addition to the SDPs reported above, we took into consideration positions that were predicted by either Xdet or S3det in both alignments (from application of ClustalO or Muscle) and which may play important structural and functional roles (Table 3 and Supplementary Information Figure S3). For example, residue Thr217 (Figure 7 and Supplementary Information Figure S3) is predicted to interact with the PLP pyridine ring. In non-YczR MocRs, this position is occupied by aromatic or hydrophobic residues putatively making stacking interaction with the PLP ring.

### 3.3. Analysis of Transcription Factor Binding Sites

Conservation of the wHTH domain of the YczR population suggests the conservation of the corresponding TFBSs upstream the YczE genes [19]. The nucleotide sequences intervening between the MocR and the YczE genes in the YczR SynTax set were scrutinized for the presence of a conserved pattern of nucleotides, possibly constituting a putative TFBS [19].

A distinctive feature of the GntR family is the presence of several TF subfamilies with very different binding motifs. Indeed, the few MocR regulators analyzed so far have revealed that the sequences of TFBSs are quite dissimilar between organisms from different taxonomical divisions, even within regulons involved in the same pathway, as in the case of PdxR [15]. For this reason, the genomic regions belonging to organisms from Actinobacteria, Alphaproteobacteria, and Gammaproteobacteria were analyzed separately.

After elimination of species redundancy, 58 YczR regulons remained in the Actinobacteria set and 15 in the Gammaproteobacteria (Supplementary Information Table S2), while the three regulons mentioned above constituted the Alphaproteobacteria set. The average length of the intergenic regions is 100 bp (ranging from 27 to 274 bp) and 86 bp (ranging from 54 to 90 bp) for Actinobacteria and Gammaproteobacteria sets, respectively. In the Alphaproteobacteria set, the sequence from* Sphingobium japonicum* is 629 bp long, while the other two sequences are composed of 61 and 90 nucleotides: their comparison, therefore, is affected by the length difference. In particular, the alignment of Actinobacteria promoter sequences is very fragmented, due to the great variability of sequence length, thus making the identification of conserved patterns through the sequences (data not shown) quite difficult.

**Figure 8:**
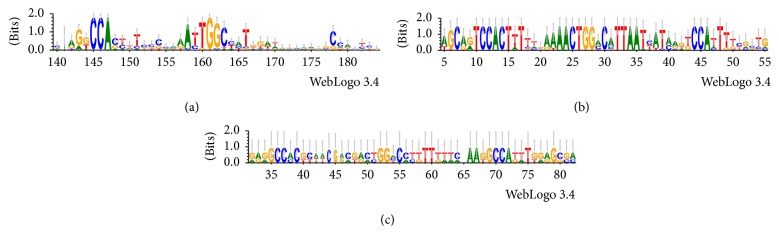
Logos of the alignments of the promoter regions. Logos of the alignments of the promoter regions from (a) Actinobacteria, (b) Gammaproteobacteria, and (c) Alphaproteobacteria regulons. The height of symbols (each one representing a nucleotide) within the stack indicates the observed frequency of the corresponding nucleotide at that position. *x*-axis indicates the sequence position in the corresponding nucleotide alignment.

**Figure 9:**
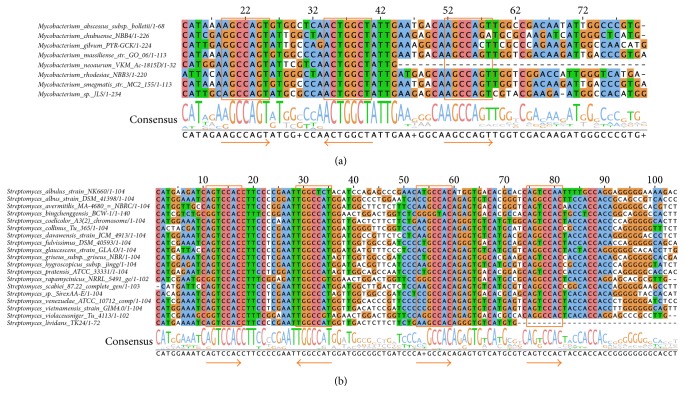
Multiple alignments of the promoter regions of the YczR regulons. Multiple alignments of the promoter regions of the YczR regulons from the Mycobacteriaceae (a) and Streptomycetaceae (b) subgroups of Actinobacteria. The DNA regions corresponding to the candidate TFBSs are highlighted by orange boxes, and the arrows indicate the direction of repeats (right for direct and left for inverted repeats). Columns are colored according to the ClustalX scheme. At the bottom of the alignments, logo of the sequences indicates the conservation of a nucleotide in the corresponding position. When more than 50% of aligned sequences have the same nucleotide in that position, the relative one-letter code is reported in the consensus row, while “+” is reported in all the other cases. For easing the interpretation of alignments, only the regions encompassing the putative TFBSs are shown.

For Actinobacteria regulons, the motif search revealed the presence of a conserved DNA motif (GGCCA) of 5 nucleotides with an inverted repeat (TGGCC) and a spacer of 12-nucleotide length (Figure 8(a)). In the Gammaproteobacteria set (Figure 8(b)), a conserved motif (GTCCACT) of 7 nucleotides with an inverted repeat (ACTGGAC), 12 bp spacing, and a directed repeat (GTCCATT) was found: both repeats present a mismatch at sixth nucleotide position of the first motif across the sequences. Interestingly, the same motifs were found in the 3 regulons belonging to the Alphaproteobacteria set (Figure 8(c)), although it should be noted that the latter logo was built from the alignment of only three promoter regions. Moreover, the logos suggest that 4 out of 5 nucleotides of the Actinobacteria motif are shared with those from the other sets. However, the logo obtained for the Actinobacteria sequences indicates that several nucleotides (around position 180) appear to be partially conserved. To test whether the detection of an additional third motif also in the Actinobacteria set was hampered by the sequence variability, two subalignments of the most represented Actinobacteria family were extracted. Alignments obtained (Figure 9) show indeed the presence of an additional directed repeat sequence of 7 nucleotides, for Mycobacteriaceae and Streptomycetaceae regulons. Furthermore, the Streptomycetaceae set shows a fourth additional direct repeat, although with mismatches with respect to the first ones.

The search of TFBSs in the databases of known DNA-binding motifs integrated in the MEME webserver (Prodoric and RegTransBase), using as a query the single repetition and a consensus sequence of the three repetitions derived from the original alignments, did not reveal any significant match with known motifs. To prove uniqueness of candidate TFBSs through the genomes, we selected three representative organisms:* Amycolatopsis mediterranei U32, Klebsiella pneumoniae *subsp.* pneumoniae MGH 78578, *and* Sphingobium chlorophenolicum L-1 *from Actinobacteria, Gammaproteobacteria, and Alphaproteobacteria, respectively. The noncoding regions of their genomes (including both forward and reverse strand) were scanned with a score matrix derived from the alignment of the sequences from all datasets. For none of the three genomes, a significant motif was detected: only the noncoding regions intervening in the predicted YczR regulons showed a significant *p* value (<0.05).

## 4. Discussion

GntR regulators are a relatively new and still poorly characterized family of transcription factors. Among them, the MocR subfamily is particularly interesting because of its homology to fold type-I PLP dependent enzymes and its role in the expression of genes involved in several metabolisms or in membrane transport of substrates. Since the last decade, this complex subfamily has been actively investigated but it is still far from being characterized and understood.

In this report, we suggest that a group of MocR regulators, predicted to regulate the expression of the uncharacterized membrane proteins YczE, can be distinguished within this subfamily by the presence of characteristic SDPs. We first confirmed that the wHTH and AAT domains of the MocR regulators, predicted by the RegPrecise databank to regulate YczE genes, cluster in the same subtree of a UPGMA cladogram built upon a multiple sequence alignment containing also MocRs from Actinobacteria and Gammaproteobacteria extracted from the same databank. Syntenic genes were afterwards identified and collected from the SynTax databank. The sequences of the MocR proteins were multiply aligned along with those previously retrieved from RegPrecise databank. Once more, both domains of the syntenic MocRs clustered in the same subtree. This pattern strongly supports the notion that the MocRs possibly involved in regulation of YczE genes share structural similarities that distinguish them from the other components of the same subfamily putatively involved in the regulation of other genes, such as those responsible for PLP biosynthesis. The YczE genes associated with divergently transcribed MocRs occur mainly in Actinobacteria and in Gammaproteobacteria. Only a few instances are observed in Alphaproteobacteria. YczE genes are widespread among bacteria and only a subpopulation is divergently transcribed with respect to MocR. Very little is known about the structure and function of YczE membrane proteins. It has been hypothesized that a YczE protein from* Bacillus subtilis B3* may belong to the ABC transport system within an operon involved in the biosynthesis of the antibiotic surfactin [45]. More recently, it has been suggested that YczE from* Bacillus amyloliquefaciens FZB42* takes part in the regulation of the biosynthesis of the iturin antibiotic bacillomycin D [46].

Application of protocols to detect SDPs and inspection of the logos pointed up residues potentially playing specific structural roles in YczR wHTH and AAT domains. In the wHTH domain, several residues are putatively involved in DNA interaction. In particular, Glu28 was identified as the discriminant residue of the YczR group by the JDet analysis. This residue, putatively interacting with the DNA phosphate backbone, precedes the “universally” conserved Arg29 (corresponding to FadR Arg35 responsible for interaction with G base in the DNA major groove). Other residues are potentially involved in DNA binding (Table 3). The comparison of regulons across multiple genomes from the same taxonomic group allows reliable prediction of TFBSs. This fact has its basis on the assumption that functional DNA sequences (such as TFBSs) diverge more slowly than the nonfunctional ones [47] (e.g., spacers in the intergenic regions). However, it should be noted that motifs similar to those discussed in this work are reported in the RegPrecise database, although in a smaller number of regulons. Moreover, subalignments of intergenic regions from Mycobacteriaceae and Streptomycetaceae regulons revealed the presence of an additional third motif (similar to the one of Alpha- and Gammaproteobacteria), not identified with the complete Actinobacteria set and not reported in databases. Noteworthily, the analysis of the upstream sequences of YczE genes has revealed the conservation of a conserved motif among all the promoter sequences examined. This observation suggests that the motifs discovered could be the DNA-binding sites of MocRs involved in YczE regulation. In particular, the presence of two directed repeats and one inverted repeat is consistent with the previous observation reported for the PdxR regulator [15]. The presence of three motifs supports, at least in the case of PdxR from* B. clausii*, the existence of two different conformations of the regulators able to selectively bind direct or inverted repeats. Noteworthily, the motifs from the three different taxonomical groups share a substantial number of conserved nucleotides. This is an exception among the MocR TFBSs investigated so far: indeed, an unequivocal and conserved TFBS motif shared by the MocR family has not been found yet [48]. This result is coherent with those obtained from the analysis of the YczR wHTH domain sequences, which cluster in the same group regardless of taxonomical origin, and consequently suggests that similarity of wHTH sequences may reflect similarity of TFBS motif recognized.

The analysis on the AAT domains of the YczR pointed several residues characteristics of this group (Table 3). Particularly interesting may be the residues Ala9 and Arg491 (Figure 7) that take part in the formation of the active site mouth. It may be speculated that these residues confer to the AAT domain specificity for effectors involved in controlling expression of YczE genes. The residue Glu215 replacing the Asp of the characteristic motif Glu-Asp-Asp occurring in most of the MocR AAT domains studied, for example, in those from Firmicutes [17], should be noted also. Although not predicted by the consensus of the JDet programs, the position 214 that in YczR is occupied almost always by a Val and in the other MocRs by a Glu should not be overlooked. Therefore, the characteristic motif Glu214-Asp215-Asp216, in the case of YczR, is better described by Val-Asp-Glu. This sequence forms part of the active site floor and therefore can be expected to influence its properties. Furthermore, residue Thr217 interacts with the PLP cofactor pyridine ring. In non-YczE MocRs, this position is occupied by an aromatic or hydrophobic residue. The presence of Thr may indeed influence reactivity of PLP. In fact, one of the open questions about MocRs is to determine whether they possess a residual enzymatic activity. In a few cases (e.g., [7]), it has been experimentally demonstrated that they do not possess significant catalytic activity. Active site inspection of the homology model and its comparison to the active site of the template suggest that many of the residues involved in cofactor or substrate interaction are conserved (Supplementary Information Figure S3). This fact may support the hypothesis that this group of regulators preserves ability to bind PLP, possibly with altered affinity and perhaps some residual catalytic activity.

## 5. Conclusions

The information available in the RegPrecise database prompted the analysis of the structural features of a MocR subgroup predicted to regulate the expression of membrane proteins of the family YczE with unknown function. We concluded that this MocR subgroup possesses distinguishing characteristics. Indeed, the wHTH and AAT domains display distinctive conserved positions which may be related to their specific functional properties. A subset of the YczE membrane protein populations is predicted to be under the control of regulators putatively able to respond to PLP and, very likely, amino acid binding. These considerations suggest that YczE proteins may be involved in transportation (influx or efflux) of metabolites, in particular amino acids, connected to pathways in which PLP takes somehow part. Indeed, YczE gene of* Bacillus amyloliquefaciens* FZB42 is adjacent to genes coding for components of an ABC transporter system predicted to be involved in polar amino acid translocation [49] within the biosynthetic process of bacteriomycin D [46]. Likewise, YczE gene in* Bacillus subtilis* strain B3 has been indicated as a potential ABC transporter component although it lacks the corresponding sequence signature [45]. All these observations prompt the rational design of experiments aimed at the characterization of YczR regulators and YczE proteins. In general, all of the results reported in this work can be tested by site directed mutagenesis. Therefore, this work can represent a framework for rationalization of experimental results and for bioinformatics analysis of other MocR subgroups.

## Supplementary Material

The Supplementary Information contains data which could not be included in the main text because of their size. In particular: Sequence alignments among YczR and non-YczR wHTH and AAT domains; the alignments contains 254 and 281 sequences with average length of 67 and 358 residues, respectively. The alignments can only be visualized on the computer screen using a PDF reader program. The figures display the conserved positions and the position characteristic of the YczR subset compared to those of the non-YczR set; A stereo picture of the AAT domain of a homology model of a YczR displaying the predicted specificity-determining positions (SDP); A table listing the non-YczR regulators taken from the RegPrecise data bank; A table listing the YczR regulators found through the SynTax analysis; A table listing the putative organisms containing the YczR regulons used to derive the DNA binding motif. More details are described in the captions to the figures included in the Supplementary Information file.

## Figures and Tables

**Table 1:** List of MocR regulators involved in YczE-containing regulons extracted from the RegPrecise databank (YczR RegPrecise set).

RefSeq code	*Specie*	*Phylum*	*RegPrecise Regulog*
WP_003977408	Multispecies:* Streptomyces *sp.	Actinobacteria	PdxR3-Streptomycetaceae
WP_013005094	*Streptomyces scabiei*	Actinobacteria	PdxR3-Streptomycetaceae
WP_010988326	*Streptomyces avermitilis*	Actinobacteria	PdxR3-Streptomycetaceae
WP_012381719	*Streptomyces griseus*	Actinobacteria	PdxR3-Streptomycetaceae
WP_010982418	*Streptomyces avermitilis*	Actinobacteria	PdxR3-Streptomycetaceae
WP_012380525	*Streptomyces griseus*	Actinobacteria	PdxR3-Streptomycetaceae
WP_015748596	*Nakamurella multipartita*	Actinobacteria	PdxR3-Frankineae/Propionibacterineae/Pseudonocardiaceae
WP_009946170	*Saccharopolyspora erythraea*	Actinobacteria	PdxR3-Frankineae/Propionibacterineae/Pseudonocardiaceae
WP_015805463	*Actinosynnema mirum*	Actinobacteria	PdxR3-Frankineae/Propionibacterineae/Pseudonocardiaceae
WP_011756808	*Nocardioides *sp.* JS614*	Actinobacteria	PdxR3-Frankineae/Propionibacterineae/Pseudonocardiaceae
WP_015884402	*Beutenbergia cavernae*	Actinobacteria	PdxR3-Micrococcineae
WP_012037448	*Clavibacter michiganensis*	Actinobacteria	PdxR3-Micrococcineae
WP_009775507	*Janibacter *sp.* HTCC2649*	Actinobacteria	PdxR3-Micrococcineae
WP_011773522	*Arthrobacter aurescens*	Actinobacteria	PdxR3-Micrococcineae
WP_015935970	*Arthrobacter chlorophenolicus*	Actinobacteria	PdxR3-Micrococcineae
WP_011690487	*Arthrobacter *sp.* FB24*	Actinobacteria	PdxR3-Micrococcineae
WP_011727370	*Mycobacterium smegmatis*	Actinobacteria	PdxR3-Mycobacteriaceae
WP_011895119	*Mycobacterium gilvum*	Actinobacteria	PdxR3-Mycobacteriaceae
WP_011779313	*Mycobacterium vanbaalenii*	Actinobacteria	PdxR3-Mycobacteriaceae
WP_019749471	*Rhodococcus*	Actinobacteria	PdxR3-Nocardiaceae
WP_011597726	*Rhodococcus jostii*	Actinobacteria	PdxR3-Nocardiaceae
WP_012523660	*Phenylobacterium zucineum*	Alphaproteobacteria	PdxR3-Caulobacterales
WP_009801668	*Oceanicaulis* sp.* HTCC2633*	Alphaproteobacteria	PdxR3-Rhodobacterales
WP_012133523	*Citrobacter koseri*	Gammaproteobacteria	PdxR3-Enterobacteriales
WP_012016330	*Enterobacter *sp.* 638*	Gammaproteobacteria	PdxR3-Enterobacteriales
WP_012068366	*Klebsiella pneumoniae*	Gammaproteobacteria	PdxR3-Enterobacteriales

**Table 2:** SDPs found in the wHTH domain of YczR and comparison with equivalent residues in MocR/other, GabR, and FadR.

YczR	MocR/other	GabR	FadR	Function in FadR
Leu12	Mostly polar residues	Lys26	Glu18	Exposed; not involved in DNA interaction
Glu28	Thr, Ser, Asn, Val, Leu, Ile, His, Gln	Lys42	Glu34	In proximity of the phosphate backbone in the DNA major groove
Thr40	Mostly Asn, Gly, Ser	Asn54	Thr46	At interaction distance with bases of the major groove; electrostatic binds with Arg35, Arg45, Arg49
Arg50	Mostly apolar; Glu, Arg	Leu64	Ala56	Interaction with the *β*-sheets of the “wing”
Ser63	Mostly Thr	Phe77	Thr69	In the *β*-sheets of the “wing”; in proximity of the phosphate backbone of DNA
Pro68	All residues	Leu82	Phe74	Interaction with the effector/oligomerization domain

**Table 3:** SDPs found in the AAT domain of YczR and comparison with the residues occurring in the MocR/other. Putative functions are attributed to homology modelling.

YczR^(a)^	MocR/other	Putative structural function
**Ala9**	Gly; large hydrophobic	At the active site mouth
**Tyr63**	Hydrophobic	Exposed to the solvent
**Asp/Glu181**	Mostly Ala, Leu, Asn, Thr	Buried; possibly interacting with Arg154
**Val213**	Glu	Buried; at C-side of Asp214 interacting with pyridine nitrogen of PLP
**Glu215**	Asp	Buried; possibly interacting with Arg154; next to the Asp214 interacting with pyridine nitrogen of PLP
**Gly394**	Ala, Gly	In a loop
**Arg491**	Val, Arg	Points to the active site; at about 7 Å distance from the phenolic oxygen of cofactor; with Ala9, forms part of the active site mouth
**Pro493**	Asn, Gly, Ser	At the C-terminal end of the *β*-sheet containing Arg491
*Pro70*	Mainly polar residues	Exposed in a loop on the opposite side of active site
*Thr71*	Polar and apolar residues; indels	In a loop; possible interaction with Arg 216
*Trp151*	Pro, hydrophobic residues	Interface between *α*-helices and inner *β*-sheet of the major domain
*Thr217*	Hydrophobic or aromatic	Stacking with the cofactor pyridine ring
*Trp296*	Polar and apolar residues; in a few cases, Trp also	In the loop containing the aldimine-forming lysine
*Arg216*	Mainly hydrophobic	Exposed; possible interaction with Thr71

^(a)^Boldfaced residues correspond to SDPs accepted by consensus approach (see text); italicized residues are SDPs predicted by either Xdet or S3det in both alignments.
