# Supplementary material for: A Bioinformatics Analysis Reveals a Group of MocR Bacterial Transcriptional Regulators Linked to a Family of Genes Coding for Membrane Proteins
Source: Biochem Res Int. 2016 Jun 30;2016:4360285. doi: 10.1155/2016/4360285 (PMC4944035; doi:10.1155/2016/4360285)
Supplement: Supplementary file 1 — The Supplementary Information contains data which could not be included in the main text because of their size. In particular: Sequence alignments among YczR and non-YczR wHTH and AAT domains; the alignments contains 254 and 281 sequences with average length of 67 and 358 residues, respectively. The alignments can only be visualized on the computer screen using a PDF reader program. The figures display the conserved positions and the position characteristic of the YczR subset compared to those of the non-YczR set; A stereo picture of the AAT domain of a homology model of a YczR displaying the predicted specificity-determining positions (SDP); A table listing the non-YczR regulators taken from the RegPrecise data bank; A table listing the YczR regulators found through the SynTax analysis; A table listing the putative organisms containing the YczR regulons used to derive the DNA binding motif. More details are described in the captions to the figures included in the Supplementary Information file. [file 4360285.f1.pdf]

Supplementary Information to:

A bioinformatics analysis reveals a group of MocR bacterial transcriptional regulators linked to a family of genes coding for membrane proteins

Teresa Milano[1], Sebastiana Angelaccio[1], Angela Tramonti[2,1], Martino Luigi Di Salvo[1], Roberto Contestabile[1] and Stefano Pascarella[1,§]

[1]Dipartimento di Scienze biochimiche, Sapienza Università di Roma, 00185 Roma, Italy

[2]Istituto di Biologia e Patologia Molecolari, Consiglio Nazionale delle Ricerche, Roma, Italy

§ Corresponding author
Stefano Pascarella
Dipartimento di Scienze biochimiche
Università La Sapienza
00185 Roma
tel. +39 06 49917694
fax +39 06 49917566
e-mail: Stefano.Pascarella@uniroma1.it

Supplementary Information Figure S1

Multiple sequence alignment obtained with ClustalO of the wHTH domains of the MocR_ALL dataset filtered at 90% sequence identity. Sequences are labeled with their GenInfo and RefSeq codes. Codes on grey background indicate YczR set. Coloring code follows the ClustalX scheme.

Supplementary Information Figure S2

Multiple sequence alignment obtained with ClustalO of the AAT domains of the MocR_ALL dataset filtered at 90% sequence identity. Display is as in Supporting Information Figure 1.

Supplementary Information Figure S3

Stereo picture of the residues indicated by the software JDet as potential SDPs in the homology model of the AAT domain of the MocR from *Saccharopolyspora erithraea.* Magenta stick models correspond to residues already reported in main text Fig. 7; SDPs predicted by either Xdet or S3det in both alignments and which may play important structural and functional roles are indicated with grey stick models (italicized in main text Table 4). Numbering refers to manuscript Figure 6. Panel B displays the model structure rotated by about 180° along the Y axis with respect to representation in panel A.

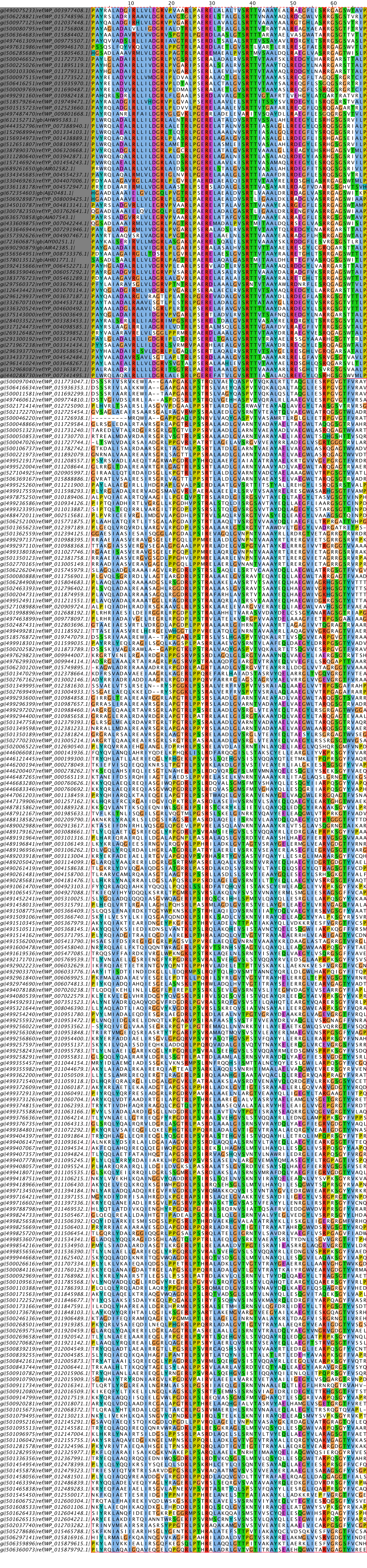
10
20
30
40
50
60

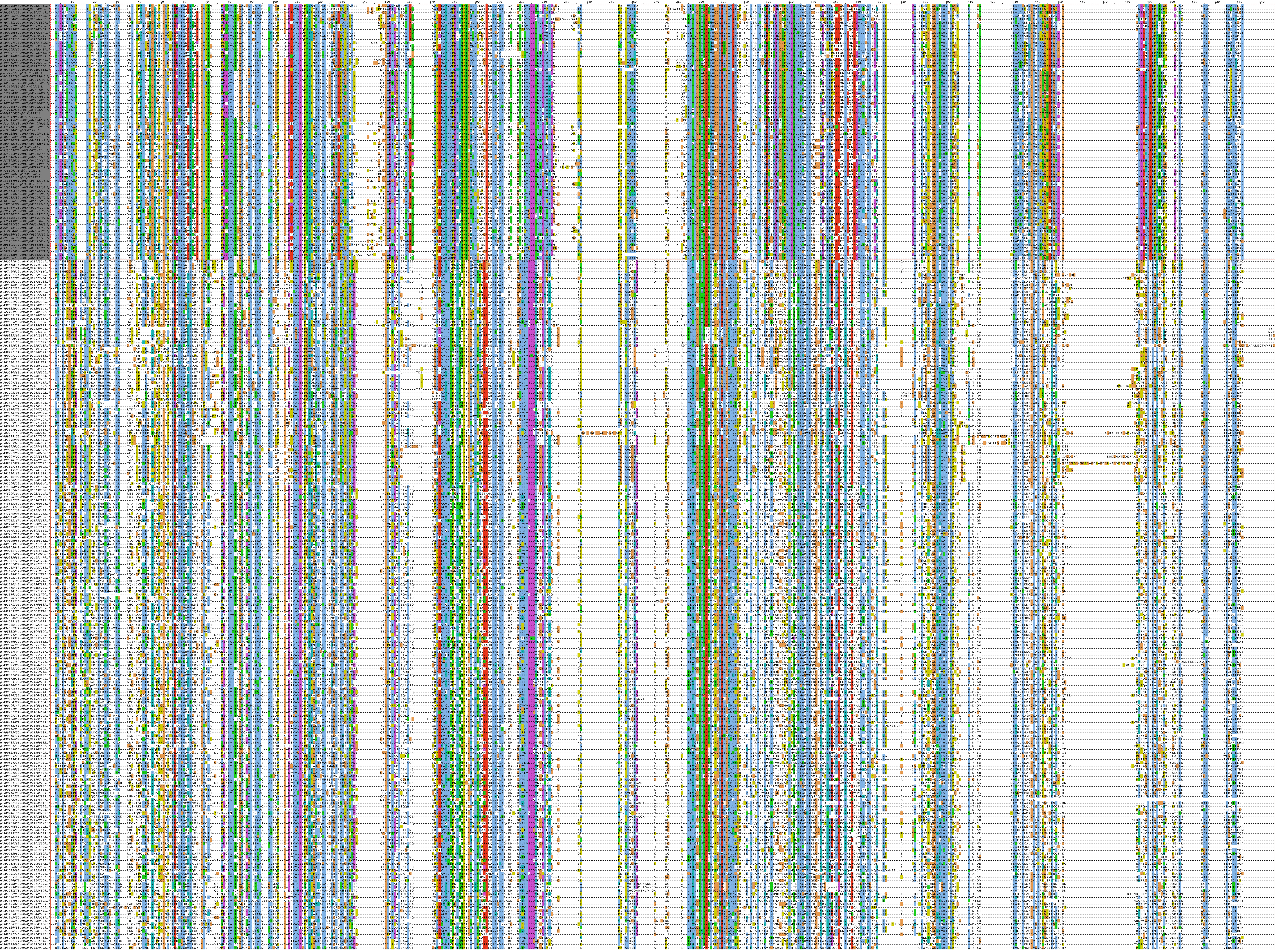

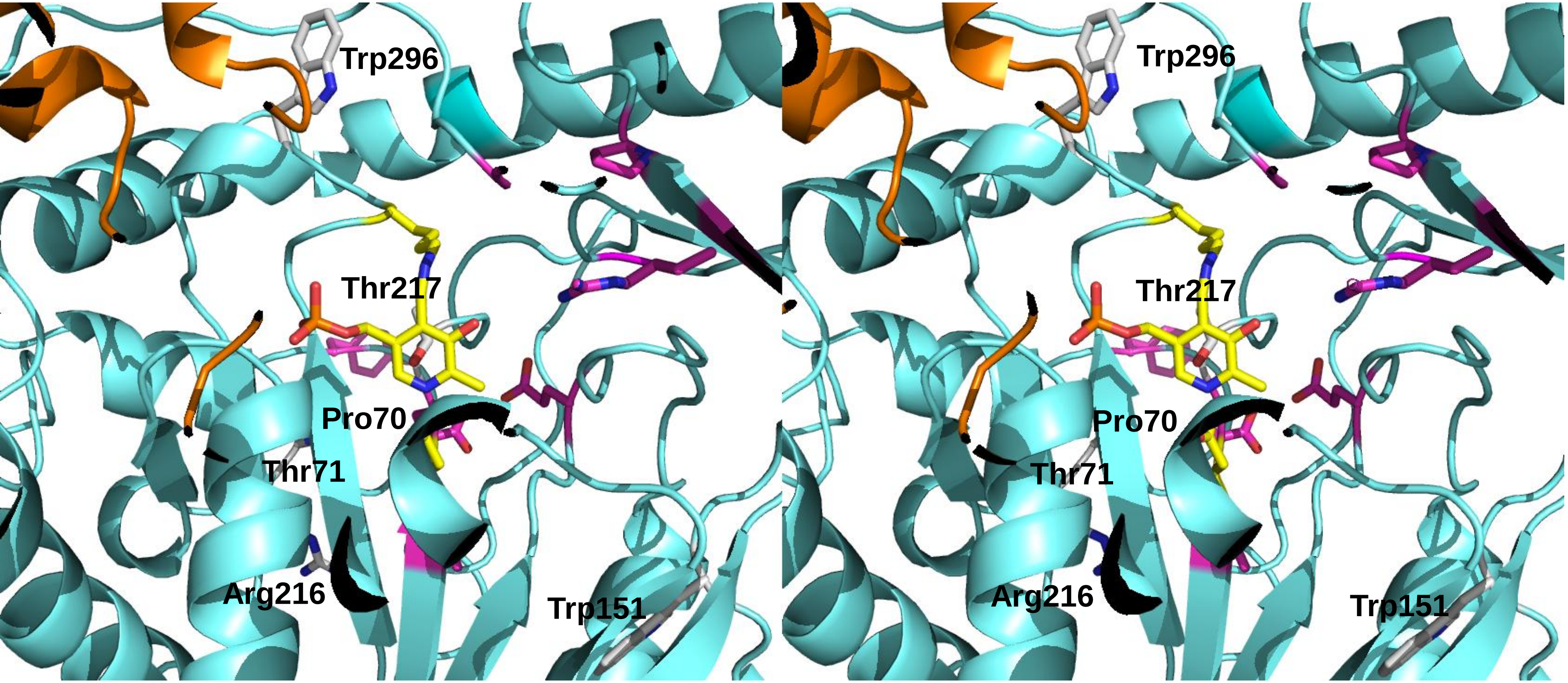

Supporting Figure S3A

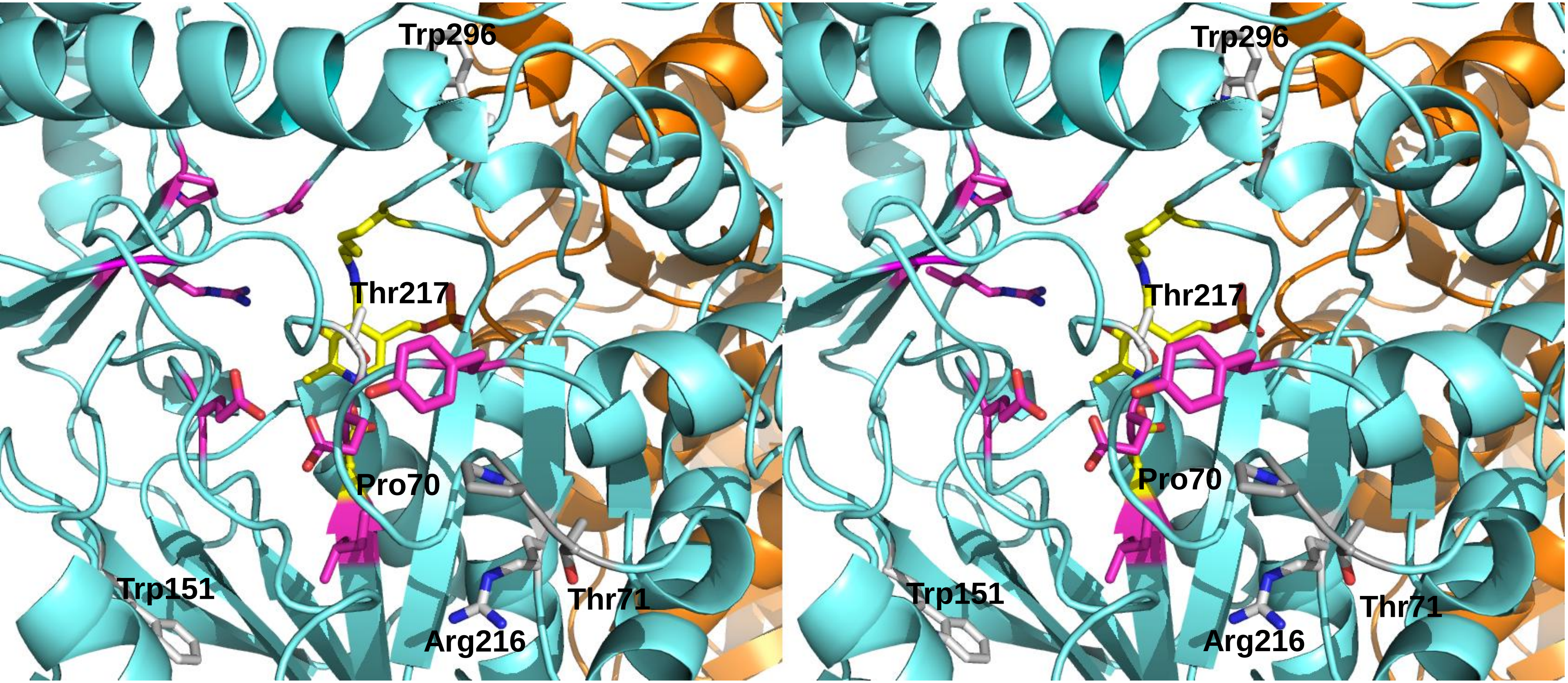

Supporting Figure S3B

Supplementary Information Table S1

List of MocR regulators not predicted to regulate DUF161-containing genes extracted from the RegPrecise data bank (MocR/other RegPrecise set).

RefSeq code	Specie	Phylum
AHW95383.1	*Enterobacter asburiae L1*	*Gammaproteobacteria*
YP_007388940.1	*Enterobacter aerogenes EA1509E*	*Gammaproteobacteria*
YP_004592771.1	*Enterobacter aerogenes KCTC 2190*	*Gammaproteobacteria*
YP_001334103.1	*Klebsiella pneumoniae subsp. pneumoniae MGH 78578*	*Gammaproteobacteria*
YP_005953387.1	*Klebsiella pneumoniae KCTC 2242*	*Gammaproteobacteria*
AJB33817.1	*Klebsiella pneumoniae HK787*	*Gammaproteobacteria*
YP_003611728.1	*Enterobacter cloacae subsp. cloacae ATCC 13047*	*Gammaproteobacteria*
YP_004827492.1	*Enterobacter asburiae LF7a*	*Gammaproteobacteria*
AHE45995.1	*Klebsiella pneumoniae subsp. pneumoniae Kp13*	*Gammaproteobacteria*
YP_007845935.1	*Enterobacter cloacae subsp. cloacae NCTC 9394*	*Gammaproteobacteria*
YP_002918142.1	*Klebsiella pneumoniae subsp. pneumoniae NTUH-K2044*	*Gammaproteobacteria*
YP_006637640.1	*Klebsiella pneumoniae subsp. pneumoniae 1084*	*Gammaproteobacteria*
AIK80736.1	*Klebsiella pneumoniae subsp. pneumoniae*	*Gammaproteobacteria*
YP_005225463.1	*Klebsiella pneumoniae subsp. pneumoniae HS11286*	*Gammaproteobacteria*
YP_008426895.1	*Klebsiella pneumoniae JM45*	*Gammaproteobacteria*
AHI34646.1	*Klebsiella pneumoniae ATCC BAA-2146*	*Gammaproteobacteria*
AHM81002.1	*Klebsiella pneumoniae 30684/NJST258_2*	*Gammaproteobacteria*
AHM86676.1	*Klebsiella pneumoniae 30660/NJST258_1*	*Gammaproteobacteria*
YP_003440871.1	*Klebsiella variicola At-22*	*Gammaproteobacteria*
YP_002240059.1	*Klebsiella pneumoniae 342*	*Gammaproteobacteria*
YP_006476484.1	*Enterobacter cloacae subsp. dissolvens SDM*	*Gammaproteobacteria*
AHW90325.1	*Klebsiella oxytoca HKOPL1*	*Gammaproteobacteria*
YP_005018537.1	*Klebsiella oxytoca KCTC 1686*	*Gammaproteobacteria*
YP_006497050.1	*Klebsiella oxytoca E718*	*Gammaproteobacteria*
YP_007874856.1	*Raoultella ornithinolytica B6*	*Gammaproteobacteria*
YP_001438889.1	*Cronobacter sakazakii ATCC BAA-894*	*Gammaproteobacteria*
YP_006343997.1	*Cronobacter sakazakii ES15*	*Gammaproteobacteria*
YP_007441558.1	*Cronobacter sakazakii SP291*	*Gammaproteobacteria*
YP_001175660.1	*Enterobacter sp. 638*	*Gammaproteobacteria*
YP_008897761.1	*Cronobacter sakazakii CMCC 45402*	*Gammaproteobacteria*
YP_006577561.1	*Enterobacter cloacae subsp. cloacae ENHKU01*	*Gammaproteobacteria*

YP_004950843.1	*Enterobacter cloacae EcWSU1*	*Gammaproteobacteria*
AIA40830.1	*Klebsiella pneumoniae subsp. pneumoniae KPNIH27*	*Gammaproteobacteria*
AIV28670.1	*Enterobacter cloacae*	*Gammaproteobacteria*
AHE71545.1	*Enterobacter cloacae P101*	*Gammaproteobacteria*
YP_008659001.1	*Klebsiella pneumoniae CG43*	*Gammaproteobacteria*
AIE62943.1	*Enterobacter cloacae ECNIH2*	*Gammaproteobacteria*
AIG83319.1	*Klebsiella pneumoniae subsp. pneumoniae PittNDM01*	*Gammaproteobacteria*
AIA35468.1	*Klebsiella pneumoniae subsp. pneumoniae KPNIH10*	*Gammaproteobacteria*
AID98003.1	*Klebsiella pneumoniae subsp. pneumoniae KPNIH24*	*Gammaproteobacteria*
AIE21782.1	*Klebsiella pneumoniae subsp. pneumoniae KPNIH1*	*Gammaproteobacteria*
AIE27161.1	*Klebsiella pneumoniae subsp. pneumoniae KPR0928*	*Gammaproteobacteria*
KEF78156.1	*Klebsiella pneumoniae UHKPC45*	*Gammaproteobacteria*
AIE02062.1	*Klebsiella pneumoniae subsp. pneumoniae KP5-1*	*Gammaproteobacteria*
AIN27052.1	*Enterobacter cloacae ECR091*	*Gammaproteobacteria*
AIN21709.1	*Enterobacter cloacae ECNIH3*	*Gammaproteobacteria*
AIE69070.1	*Klebsiella oxytoca*	*Gammaproteobacteria*
AID88783.1	*Klebsiella oxytoca KONIH1*	*Gammaproteobacteria*
YP_008109897.1	*Enterobacter sp. R4-368*	*Gammaproteobacteria*
YP_006320668.1	*Shimwellia blattae DSM 4481 = NBRC 105725*	*Gammaproteobacteria*
YP_003942871.1	*Enterobacter lignolyticus SCF1*	*Gammaproteobacteria*
YP_003209418.1	*Cronobacter turicensis z3032*	*Gammaproteobacteria*
YP_001454243.1	*Citrobacter koseri ATCC BAA-895*	*Gammaproteobacteria*
AIR02582.1	*Pluralibacter gergoviae*	*Gammaproteobacteria*
AHY12291.1	*Citrobacter freundii CFNIH1*	*Gammaproteobacteria*
YP_002131951.1	*Phenylobacterium zucineum HLK1*	*Alphaproteobacteria*
YP_004554237.1	*Sphingobium chlorophenolicum L-1*	*Alphaproteobacteria*
YP_003543498.1	*Sphingobium japonicum UT26S*	*Alphaproteobacteria*
YP_006660698.1	*Arthrobacter sp. Rue61a*	*Actinobacteria*
YP_946574.1	*Arthrobacter aurescens TC1*	*Actinobacteria*
YP_830119.1	*Arthrobacter sp. FB24*	*Actinobacteria*
YP_002486834.1	*Arthrobacter chlorophenolicus A6*	*Actinobacteria*
YP_004407006.1	*Verrucosispora maris AB-18-032*	*Actinobacteria*
AJF64107.1	*Streptomyces vietnamensis*	*Actinobacteria*
YP_007858151.1	*Streptomyces sp. PAMC26508*	*Actinobacteria*
YP_004926369.1	*Streptomyces pratensis ATCC 33331*	*Actinobacteria*

YP_006876554.1	*Streptomyces venezuelae ATCC 10712*	*Actinobacteria*
YP_004572947.1	*Microlunatus phosphovorus NM-1*	*Actinobacteria*
YP_001109088.1	*Saccharopolyspora erythraea NRRL 2338*	*Actinobacteria*
AIJ20481.1	*Amycolatopsis methanolica 239*	*Actinobacteria*
YP_008009425.1	*Amycolatopsis orientalis HCCB10007*	*Actinobacteria*
AIG80292.1	*Amycolatopsis japonica*	*Actinobacteria*
YP_003493098.1	*Streptomyces scabiei 87.22*	*Actinobacteria*
YP_003837530.1	*Micromonospora aurantiaca ATCC 27029*	*Actinobacteria*
YP_004083524.1	*Micromonospora sp. L5*	*Actinobacteria*
YP_004813141.1	*Streptomyces violaceusniger Tu 4113*	*Actinobacteria*
YP_007525677.1	*Streptomyces davawensis JCM 4913*	*Actinobacteria*
YP_001827627.1	*Streptomyces griseus subsp. griseus NBRC 13350*	*Actinobacteria*
YP_003762641.1	*Amycolatopsis mediterranei U32*	*Actinobacteria*
YP_008456750.1	*Amycolatopsis mediterranei RB*	*Actinobacteria*
YP_006546898.1	*Amycolatopsis mediterranei S699*	*Actinobacteria*
YP_007929801.1	*Streptomyces fulvissimus DSM 40593*	*Actinobacteria*
AIA07543.1	*Streptomyces albulus*	*Actinobacteria*
YP_007042244.1	*Saccharothrix espanaensis DSM 44229*	*Actinobacteria*
YP_004960546.1	*Streptomyces bingchenggensis BCW-1*	*Actinobacteria*
YP_008788370.1	*Streptomyces rapamycinicus NRRL 5491*	*Actinobacteria*
NP_828105.1	*Streptomyces avermitilis MA-4680 = NBRC 14893*	*Actinobacteria*
YP_007291946.1	*Mycobacterium smegmatis JS623*	*Actinobacteria*
YP_004907467.1	*Kitasatospora setae KM-6054*	*Actinobacteria*
YP_006243999.1	*Streptomyces hygroscopicus subsp. jinggangensis 5008*	*Actinobacteria*
YP_004801302.1	*Streptomyces sp. SirexAA-E*	*Actinobacteria*
YP_007691616.1	*Streptomyces hygroscopicus subsp. jinggangensis TL01*	*Actinobacteria*
YP_007748739.1	*Streptomyces albus J1074*	*Actinobacteria*
AIY00251.1	*Arthrobacter sp. PAMC25486*	*Actinobacteria*
AIR42385.1	*Mycobacterium abscessus subsp. bolletii*	*Actinobacteria*
AJE86892.1	*Streptomyces albus*	*Actinobacteria*
YP_008733376.1	*Actinoplanes friuliensis DSM 7358*	*Actinobacteria*
AHI01771.1	*Kutzneria albida DSM 43870*	*Actinobacteria*
YP_001538203.1	*Salinispora arenicola CNS-205*	*Actinobacteria*
YP_003104432.1	*Actinosynnema mirum DSM 43827*	*Actinobacteria*
YP_006452271.1	*Mycobacterium chubuense NBB4*	*Actinobacteria*

YP_006057292.1	*Streptomyces cattleya NRRL 8057 = DSM 46488*	*Actinobacteria*
YP_008385100.1	*Streptomyces collinus Tu 365*	*Actinobacteria*
NP_625699.1	*Streptomyces coelicolor A3(2)*	*Actinobacteria*
AIJ17027.1	*Streptomyces lividans TK24*	*Actinobacteria*
YP_001159996.1	*Salinispora tropica CNB-440*	*Actinobacteria*
YP_008906570.1	*Mycobacterium neoaurum VKM Ac-1815D*	*Actinobacteria*
AIU12919.1	*Mycobacterium smegmatis*	*Actinobacteria*
YP_952905.1	*Mycobacterium vanbaalenii PYR-1*	*Actinobacteria*
YP_006565789.1	*Mycobacterium smegmatis str. MC2 155*	*Actinobacteria*
YP_001135532.1	*Mycobacterium gilvum PYR-GCK*	*Actinobacteria*
YP_005461289.1	*Actinoplanes missouriensis 431*	*Actinobacteria*
AIR97161.1	*Streptomyces glaucescens*	*Actinobacteria*
YP_004078064.1	*Mycobacterium gilvum Spyr1*	*Actinobacteria*
YP_003679346.1	*Nocardiopsis dassonvillei subsp. dassonvillei DSM 43111*	*Actinobacteria*
YP_001070114.1	*Mycobacterium sp. JLS*	*Actinobacteria*
YP_639019.1	*Mycobacterium sp. MCS*	*Actinobacteria*
YP_937891.1	*Mycobacterium sp. KMS*	*Actinobacteria*
YP_009038400.1	*Mycobacterium abscessus subsp. bolletii str. GO 06*	*Actinobacteria*
YP_003637187.1	*Cellulomonas flavigena DSM 20109*	*Actinobacteria*
YP_004453718.1	*Cellulomonas fimi ATCC 484*	*Actinobacteria*
YP_008023793.1	*Mycobacterium abscessus subsp. bolletii 50594*	*Actinobacteria*
YP_004600492.1		*Actinobacteria*
YP_001704117.1	*Mycobacterium abscessus*	*Actinobacteria*
CAM63463.1	*Mycobacterium abscessus ATCC 19977*	*Actinobacteria*
AIV12145.1	*Mycobacterium abscessus subsp. bolletii*	*Actinobacteria*
AIR34547.1	*Mycobacterium abscessus subsp. bolletii 103*	*Actinobacteria*
YP_005003649.1	*Mycobacterium rhodesiae NBB3*	*Actinobacteria*
YP_001221503.1	*Clavibacter michiganensis subsp. michiganensis NCPPB 382*	*Actinobacteria*
YP_001709783.1	*Clavibacter michiganensis subsp. sepedonicus*	*Actinobacteria*
YP_006643068.1	*Nocardiopsis alba ATCC BAA-2165*	*Actinobacteria*
YP_007685022.1	*Clavibacter michiganensis subsp. nebraskensis NCPPB 2581*	*Actinobacteria*
YP_003383453.1	*Kribbella flavida DSM 17836*	*Actinobacteria*
YP_004098585.1	*Intrasporangium calvum DSM 43043*	*Actinobacteria*
YP_002883927.1	*Beutenbergia cavernae DSM 12333*	*Actinobacteria*
YP_003299852.1	*Thermomonospora curvata DSM 43183*	*Actinobacteria*

YP_924561.1	*Nocardioides sp. JS614*	*Actinobacteria*
YP_003511470.1	*Stackebrandtia nassauensis DSM 44728*	*Actinobacteria*
YP_003202719.1	*Nakamurella multipartita DSM 44233*	*Actinobacteria*
YP_003341434.1	*Streptosporangium roseum DSM 43021*	*Actinobacteria*
YP_003658654.1	*Segniliparus rotundus DSM 44985*	*Actinobacteria*
YP_004542484.1	*Isoptericola variabilis 225*	*Actinobacteria*
YP_003648267.1	*Tsukamurella paurometabola DSM 20162*	*Actinobacteria*
YP_001363871.1	*Kineococcus radiotolerans SRS30216 = ATCC BAA-149*	*Actinobacteria*

Supplementary information Table S2

List of YczR extracted from the SynTax data bank (YczR SynTax).

RefSeq code	Specie	Phylum
AHW95383.1	*Enterobacter asburiae L1*	*Gammaproteobacteria*
YP_007388940.1	*Enterobacter aerogenes EA1509E*	*Gammaproteobacteria*
YP_004592771.1	*Enterobacter aerogenes KCTC 2190*	*Gammaproteobacteria*
YP_001334103.1	*Klebsiella pneumoniae subsp. pneumoniae MGH 78578*	*Gammaproteobacteria*
YP_005953387.1	*Klebsiella pneumoniae KCTC 2242*	*Gammaproteobacteria*
AJB33817.1	*Klebsiella pneumoniae HK787*	*Gammaproteobacteria*
YP_003611728.1	*Enterobacter cloacae subsp. cloacae ATCC 13047*	*Gammaproteobacteria*
YP_004827492.1	*Enterobacter asburiae LF7a*	*Gammaproteobacteria*
AHE45995.1	*Klebsiella pneumoniae subsp. pneumoniae Kp13*	*Gammaproteobacteria*
YP_007845935.1	*Enterobacter cloacae subsp. cloacae NCTC 9394*	*Gammaproteobacteria*
YP_002918142.1	*Klebsiella pneumoniae subsp. pneumoniae NTUH-K2044*	*Gammaproteobacteria*
YP_006637640.1	*Klebsiella pneumoniae subsp. pneumoniae 1084*	*Gammaproteobacteria*
AIK80736.1	*Klebsiella pneumoniae subsp. pneumoniae*	*Gammaproteobacteria*
YP_005225463.1	*Klebsiella pneumoniae subsp. pneumoniae HS11286*	*Gammaproteobacteria*
YP_008426895.1	*Klebsiella pneumoniae JM45*	*Gammaproteobacteria*
AHI34646.1	*Klebsiella pneumoniae ATCC BAA-2146*	*Gammaproteobacteria*
AHM81002.1	*Klebsiella pneumoniae 30684/NJST258_2*	*Gammaproteobacteria*
AHM86676.1	*Klebsiella pneumoniae 30660/NJST258_1*	*Gammaproteobacteria*
YP_003440871.1	*Klebsiella variicola At-22*	*Gammaproteobacteria*
YP_002240059.1	*Klebsiella pneumoniae 342*	*Gammaproteobacteria*
YP_006476484.1	*Enterobacter cloacae subsp. dissolvens SDM*	*Gammaproteobacteria*
AHW90325.1	*Klebsiella oxytoca HKOPL1*	*Gammaproteobacteria*
YP_005018537.1	*Klebsiella oxytoca KCTC 1686*	*Gammaproteobacteria*
YP_006497050.1	*Klebsiella oxytoca E718*	*Gammaproteobacteria*
YP_007874856.1	*Raoultella ornithinolytica B6*	*Gammaproteobacteria*
YP_001438889.1	*Cronobacter sakazakii ATCC BAA-894*	*Gammaproteobacteria*
YP_006343997.1	*Cronobacter sakazakii ES15*	*Gammaproteobacteria*
YP_007441558.1	*Cronobacter sakazakii SP291*	*Gammaproteobacteria*
YP_001175660.1	*Enterobacter sp. 638*	*Gammaproteobacteria*
YP_008897761.1	*Cronobacter sakazakii CMCC 45402*	*Gammaproteobacteria*
YP_006577561.1	*Enterobacter cloacae subsp. cloacae ENHKU01*	*Gammaproteobacteria*
YP_004950843.1	*Enterobacter cloacae EcWSU1*	*Gammaproteobacteria*

AIA40830.1	*Klebsiella pneumoniae subsp. pneumoniae KPNIH27*	*Gammaproteobacteria*
AIV28670.1	*Enterobacter cloacae*	*Gammaproteobacteria*
AHE71545.1	*Enterobacter cloacae P101*	*Gammaproteobacteria*
YP_008659001.1	*Klebsiella pneumoniae CG43*	*Gammaproteobacteria*
AIE62943.1	*Enterobacter cloacae ECNIH2*	*Gammaproteobacteria*
AIG83319.1	*Klebsiella pneumoniae subsp. pneumoniae PittNDM01*	*Gammaproteobacteria*
AIA35468.1	*Klebsiella pneumoniae subsp. pneumoniae KPNIH10*	*Gammaproteobacteria*
AID98003.1	*Klebsiella pneumoniae subsp. pneumoniae KPNIH24*	*Gammaproteobacteria*
AIE21782.1	*Klebsiella pneumoniae subsp. pneumoniae KPNIH1*	*Gammaproteobacteria*
AIE27161.1	*Klebsiella pneumoniae subsp. pneumoniae KPR0928*	*Gammaproteobacteria*
KEF78156.1	*Klebsiella pneumoniae UHKPC45*	*Gammaproteobacteria*
AIE02062.1	*Klebsiella pneumoniae subsp. pneumoniae KP5-1*	*Gammaproteobacteria*
AIN27052.1	*Enterobacter cloacae ECR091*	*Gammaproteobacteria*
AIN21709.1	*Enterobacter cloacae ECNIH3*	*Gammaproteobacteria*
AIE69070.1	*Klebsiella oxytoca*	*Gammaproteobacteria*
AID88783.1	*Klebsiella oxytoca KONIH1*	*Gammaproteobacteria*
YP_008109897.1	*Enterobacter sp. R4-368*	*Gammaproteobacteria*
YP_006320668.1	*Shimwellia blattae DSM 4481 = NBRC 105725*	*Gammaproteobacteria*
YP_003942871.1	*Enterobacter lignolyticus SCF1*	*Gammaproteobacteria*
YP_003209418.1	*Cronobacter turicensis z3032*	*Gammaproteobacteria*
YP_001454243.1	*Citrobacter koseri ATCC BAA-895*	*Gammaproteobacteria*
AIR02582.1	*Pluralibacter gergoviae*	*Gammaproteobacteria*
AHY12291.1	*Citrobacter freundii CFNIH1*	*Gammaproteobacteria*
YP_002131951.1	*Phenylobacterium zucineum HLK1*	*Alphaproteobacteria*
YP_004554237.1	*Sphingobium chlorophenolicum L-1*	*Alphaproteobacteria*
YP_003543498.1	*Sphingobium japonicum UT26S*	*Alphaproteobacteria*
YP_006660698.1	*Arthrobacter sp. Rue61a*	*Actinobacteria*
YP_946574.1	*Arthrobacter aurescens TC1*	*Actinobacteria*
YP_830119.1	*Arthrobacter sp. FB24*	*Actinobacteria*
YP_002486834.1	*Arthrobacter chlorophenolicus A6*	*Actinobacteria*
YP_004407006.1	*Verrucosispora maris AB-18-032*	*Actinobacteria*
AJF64107.1	*Streptomyces vietnamensis*	*Actinobacteria*
YP_007858151.1	*Streptomyces sp. PAMC26508*	*Actinobacteria*
YP_004926369.1	*Streptomyces pratensis ATCC 33331*	*Actinobacteria*
YP_006876554.1	*Streptomyces venezuelae ATCC 10712*	*Actinobacteria*

YP_004572947.1	*Microlunatus phosphovorus NM-1*	*Actinobacteria*
YP_001109088.1	*Saccharopolyspora erythraea NRRL 2338*	*Actinobacteria*
AIJ20481.1	*Amycolatopsis methanolica 239*	*Actinobacteria*
YP_008009425.1	*Amycolatopsis orientalis HCCB10007*	*Actinobacteria*
AIG80292.1	*Amycolatopsis japonica*	*Actinobacteria*
YP_003493098.1	*Streptomyces scabiei 87.22*	*Actinobacteria*
YP_003837530.1	*Micromonospora aurantiaca ATCC 27029*	*Actinobacteria*
YP_004083524.1	*Micromonospora sp. L5*	*Actinobacteria*
YP_004813141.1	*Streptomyces violaceusniger Tu 4113*	*Actinobacteria*
YP_007525677.1	*Streptomyces davawensis JCM 4913*	*Actinobacteria*
YP_001827627.1	*Streptomyces griseus subsp. griseus NBRC 13350*	*Actinobacteria*
YP_003762641.1	*Amycolatopsis mediterranei U32*	*Actinobacteria*
YP_008456750.1	*Amycolatopsis mediterranei RB*	*Actinobacteria*
YP_006546898.1	*Amycolatopsis mediterranei S699*	*Actinobacteria*
YP_007929801.1	*Streptomyces fulvissimus DSM 40593*	*Actinobacteria*
AIA07543.1	*Streptomyces albulus*	*Actinobacteria*
YP_007042244.1	*Saccharothrix espanaensis DSM 44229*	*Actinobacteria*
YP_004960546.1	*Streptomyces bingchenggensis BCW-1*	*Actinobacteria*
YP_008788370.1	*Streptomyces rapamycinicus NRRL 5491*	*Actinobacteria*
NP_828105.1	*Streptomyces avermitilis MA-4680 = NBRC 14893*	*Actinobacteria*
YP_007291946.1	*Mycobacterium smegmatis JS623*	*Actinobacteria*
YP_004907467.1	*Kitasatospora setae KM-6054*	*Actinobacteria*
YP_006243999.1	*Streptomyces hygroscopicus subsp. jinggangensis 5008*	*Actinobacteria*
YP_004801302.1	*Streptomyces sp. SirexAA-E*	*Actinobacteria*
YP_007691616.1	*Streptomyces hygroscopicus subsp. jinggangensis TL01*	*Actinobacteria*
YP_007748739.1	*Streptomyces albus J1074*	*Actinobacteria*
AIY00251.1	*Arthrobacter sp. PAMC25486*	*Actinobacteria*
AIR42385.1	*Mycobacterium abscessus subsp. bolletii*	*Actinobacteria*
AJE86892.1	*Streptomyces albus*	*Actinobacteria*
YP_008733376.1	*Actinoplanes friuliensis DSM 7358*	*Actinobacteria*
AHI01771.1	*Kutzneria albida DSM 43870*	*Actinobacteria*
YP_001538203.1	*Salinispora arenicola CNS-205*	*Actinobacteria*
YP_003104432.1	*Actinosynnema mirum DSM 43827*	*Actinobacteria*
YP_006452271.1	*Mycobacterium chubuense NBB4*	*Actinobacteria*
YP_006057292.1	*Streptomyces cattleya NRRL 8057 = DSM 46488*	*Actinobacteria*

YP_008385100.1	*Streptomyces collinus Tu 365*	*Actinobacteria*
NP_625699.1	*Streptomyces coelicolor A3(2)*	*Actinobacteria*
AIJ17027.1	*Streptomyces lividans TK24*	*Actinobacteria*
YP_001159996.1	*Salinispora tropica CNB-440*	*Actinobacteria*
YP_008906570.1	*Mycobacterium neoaurum VKM Ac-1815D*	*Actinobacteria*
AIU12919.1	*Mycobacterium smegmatis*	*Actinobacteria*
YP_952905.1	*Mycobacterium vanbaalenii PYR-1*	*Actinobacteria*
YP_006565789.1	*Mycobacterium smegmatis str. MC2 155*	*Actinobacteria*
YP_001135532.1	*Mycobacterium gilvum PYR-GCK*	*Actinobacteria*
YP_005461289.1	*Actinoplanes missouriensis 431*	*Actinobacteria*
AIR97161.1	*Streptomyces glaucescens*	*Actinobacteria*
YP_004078064.1	*Mycobacterium gilvum Spyr1*	*Actinobacteria*
YP_003679346.1	*Nocardiopsis dassonvillei subsp. dassonvillei DSM 43111*	*Actinobacteria*
YP_001070114.1	*Mycobacterium sp. JLS*	*Actinobacteria*
YP_639019.1	*Mycobacterium sp. MCS*	*Actinobacteria*
YP_937891.1	*Mycobacterium sp. KMS*	*Actinobacteria*
YP_009038400.1	*Mycobacterium abscessus subsp. bolletii str. GO 06*	*Actinobacteria*
YP_003637187.1	*Cellulomonas flavigena DSM 20109*	*Actinobacteria*
YP_004453718.1	*Cellulomonas fimi ATCC 484*	*Actinobacteria*
YP_008023793.1	*Mycobacterium abscessus subsp. bolletii 50594*	*Actinobacteria*
WP_013883443.1	*Cellvibrio gilvus*	*Actinobacteria*
YP_001704117.1	*Mycobacterium abscessus*	*Actinobacteria*
CAM63463.1	*Mycobacterium abscessus ATCC 19977*	*Actinobacteria*
AIV12145.1	*Mycobacterium abscessus subsp. bolletii*	*Actinobacteria*
AIR34547.1	*Mycobacterium abscessus subsp. bolletii 103*	*Actinobacteria*
YP_005003649.1	*Mycobacterium rhodesiae NBB3*	*Actinobacteria*
YP_001221503.1	*Clavibacter michiganensis subsp. michiganensis NCPPB 382*	*Actinobacteria*
YP_001709783.1	*Clavibacter michiganensis subsp. sepedonicus*	*Actinobacteria*
YP_006643068.1	*Nocardiopsis alba ATCC BAA-2165*	*Actinobacteria*
YP_007685022.1	*Clavibacter michiganensis subsp. nebraskensis NCPPB 2581*	*Actinobacteria*
YP_003383453.1	*Kribbella flavida DSM 17836*	*Actinobacteria*
YP_004098585.1	*Intrasporangium calvum DSM 43043*	*Actinobacteria*
YP_002883927.1	*Beutenbergia cavernae DSM 12333*	*Actinobacteria*
YP_003299852.1	*Thermomonospora curvata DSM 43183*	*Actinobacteria*
YP_924561.1	*Nocardioides sp. JS614*	*Actinobacteria*

YP_003511470.1	*Stackebrandtia nassauensis DSM 44728*	*Actinobacteria*
YP_003202719.1	*Nakamurella multipartita DSM 44233*	*Actinobacteria*
YP_003341434.1	*Streptosporangium roseum DSM 43021*	*Actinobacteria*
YP_003658654.1	*Segniliparus rotundus DSM 44985*	*Actinobacteria*
YP_004542484.1	*Isoptericola variabilis 225*	*Actinobacteria*
YP_003648267.1	*Tsukamurella paurometabola DSM 20162*	*Actinobacteria*
YP_001363871.1	*Kineococcus radiotolerans SRS30216 = ATCC BAA-149*	*Actinobacteria*

Supplementary information Table S3

Non redundant dataset of organisms containing putative YczR regulons used for the detection of DNA-binding motifs.

Specie	Phylum	Intergenic region lenght
Actinoplanes friuliensis DSM 7358	*Actinobacteria*	60
Actinosynnema mirum DSM 43827	*Actinobacteria*	62
Amycolatopsis japonica	*Actinobacteria*	62
Amycolatopsis mediterranei U32	*Actinobacteria*	107
Amycolatopsis methanolica 239	*Actinobacteria*	114
Amycolatopsis orientalis HCCB10007	*Actinobacteria*	62
Arthrobacter aurescens TC1	*Actinobacteria*	61
Arthrobacter chlorophenolicus A6	*Actinobacteria*	61
Arthrobacter sp. PAMC25486	*Actinobacteria*	60
Cellulomonas fimi ATCC 484	*Actinobacteria*	251
Cellvibrio gilvus	*Actinobacteria*	217
Clavibacter michiganensis subsp. michiganensis NCPPB 382	*Actinobacteria*	63
Intrasporangium calvum DSM 43043	*Actinobacteria*	63
Isoptericola variabilis 225	*Actinobacteria*	62
Kineococcus radiotolerans SRS30216 = ATCC BAA-149	*Actinobacteria*	62
Kribbella flavida DSM 17836	*Actinobacteria*	62
Kutzneria albida DSM 43870	*Actinobacteria*	62
Microlunatus phosphovorus NM-1	*Actinobacteria*	115
Micromonospora aurantiaca ATCC 27029	*Actinobacteria*	60
Micromonospora sp. L5	*Actinobacteria*	60
Mycobacterium abscessus subsp. bolletii 103	*Actinobacteria*	63
Mycobacterium chubuense NBB4	*Actinobacteria*	221
Mycobacterium gilvum PYR-GCK	*Actinobacteria*	219
Mycobacterium neoaurum VKM Ac-1815D	*Actinobacteria*	27
Mycobacterium rhodesiae NBB3	*Actinobacteria*	215
Mycobacterium smegmatis str. MC2 155	*Actinobacteria*	63
Mycobacterium sp. JLS	*Actinobacteria*	229
Mycobacterium vanbaalenii PYR-1	*Actinobacteria*	144
Nakamurella multipartita DSM 44233	*Actinobacteria*	62
Nocardioides sp. JS614	*Actinobacteria*	62
Saccharopolyspora erythraea NRRL 2338	*Actinobacteria*	62
Saccharothrix espanaensis DSM 44229	*Actinobacteria*	63
Salinispora arenicola CNS-205	*Actinobacteria*	62
Salinispora tropica CNB-440	*Actinobacteria*	62
Segniliparus rotundus DSM 44985	*Actinobacteria*	114
Stackebrandtia nassauensis DSM 44728	*Actinobacteria*	62
Streptomyces albulus	*Actinobacteria*	102
Streptomyces albus	*Actinobacteria*	114
Streptomyces avermitilis MA-4680 = NBRC 14893	*Actinobacteria*	113
Streptomyces bingchenggensis BCW-1	*Actinobacteria*	274

Streptomyces coelicolor A3(2)	*Actinobacteria*	103
Streptomyces collinus Tu 365	*Actinobacteria*	103
Streptomyces davawensis JCM 4913	*Actinobacteria*	101
Streptomyces fulvissimus DSM 40593	*Actinobacteria*	103
Streptomyces glaucescens	*Actinobacteria*	99
Streptomyces griseus subsp. griseus NBRC 13350	*Actinobacteria*	103
Streptomyces hygroscopicus subsp. jinggangensis 5008	*Actinobacteria*	104
Streptomyces lividans TK24	*Actinobacteria*	67
Streptomyces pratensis ATCC 33331	*Actinobacteria*	103
Streptomyces rapamycinicus NRRL 5491	*Actinobacteria*	97
Streptomyces scabiei 87.22	*Actinobacteria*	195
Streptomyces sp. SirexAA-E	*Actinobacteria*	100
Streptomyces venezuelae ATCC 10712	*Actinobacteria*	102
Streptomyces vietnamensis	*Actinobacteria*	104
Streptomyces violaceusniger Tu 4113	*Actinobacteria*	97
Streptosporangium roseum DSM 43021	*Actinobacteria*	49
Tsukamurella paurometabola DSM 20162	*Actinobacteria*	44
Verrucosispora maris AB-18-032	*Actinobacteria*	89
Citrobacter freundii CFNIH1	*Gammaproteobacteria*	89
Citrobacter koseri ATCC BAA-895	*Gammaproteobacteria*	87
Cronobacter sakazakii ATCC BAA-894	*Gammaproteobacteria*	87
Cronobacter turicensis z3032	*Gammaproteobacteria*	54
Enterobacter aerogenes EA1509E	*Gammaproteobacteria*	88
Enterobacter asburiae L1	*Gammaproteobacteria*	87
Enterobacter cloacae EcWSU1	*Gammaproteobacteria*	88
Enterobacter lignolyticus SCF1	*Gammaproteobacteria*	89
Enterobacter sp. 638	*Gammaproteobacteria*	85
Klebsiella oxytoca KONIH1	*Gammaproteobacteria*	88
Klebsiella pneumoniae subsp. pneumoniae	*Gammaproteobacteria*	89
Klebsiella variicola At-22	*Gammaproteobacteria*	89
Pluralibacter gergoviae	*Gammaproteobacteria*	89
Raoultella ornithinolytica B6	*Gammaproteobacteria*	93
Shimwellia blattae DSM 4481 = NBRC 105725	*Gammaproteobacteria*	90
Phenylobacterium zucineum HLK1	*Alphaproteobacteria*	61
Sphingobium chlorophenolicum L-1	*Alphaproteobacteria*	90
Sphingobium japonicum UT26S	*Alphaproteobacteria*	629
